# Light-activated semiconductor gas sensors: pathways to improve sensitivity and reduce energy consumption

**DOI:** 10.3389/fchem.2025.1538217

**Published:** 2025-02-25

**Authors:** Abulkosim Nasriddinov, Rustem Zairov, Marina Rumyantseva

**Affiliations:** ^1^ Chemistry Department, Moscow State University, Moscow, Russia; ^2^ Federal Research Center Kazan Scientific Center of Russian Academy of Sciences, Kazan, Russia

**Keywords:** semiconductor gas sensor, low operating temperature, ultraviolet/visible light illumination, photosensitive materials, light-activation, photo-irradiation, energy consumption

## Abstract

Resistive type gas sensors based on wide-bandgap semiconductor oxides are remaining one of the principal players in environmental air monitoring. The rapid development of technology and the desire to miniaturize electronics require the creation of devices with minimal energy consumption. A promising solution may be the use of photoactivation, which can initiate/accelerate physico-chemical processes at the solid-gas interface and realize detection of flammable and explosive gases at close to room temperature. This work examines the mechanism underlying the increased sensitivity to various gases under photoactivation. The review is intended to clarify the current situation in the field of light-activated gas sensors and set the vector for their further development in order to integrate with the latest technological projects.

## 1 Introduction

Over the past decades, the rapid development of industry has led to an increase in the number and type of pollutants and toxic substances in the atmosphere. The pollution level often exceeds maximum permissible concentrations in megapolices and industrial areas, which can lead to serious health problems and climate changes (World Health Organization, 2006; National Research Council, 2012; Breisinger, 2012). Therefore, the creation of air monitoring systems is an extremely important task (Santhanam and Ahamed, 2018). Currently, to determine trace gas concentrations research centers and technology laboratories use different complex methods as gas chromatography, mass spectrometry and infrared spectroscopy, which are not suitable for mass use because of the high cost of the instruments, their overall dimensions, large weight, high power consumption and complex maintenance (Wang et al., 2023). An alternative could be the development of miniature sensors for express and mobile gas detection (White and Turner, 1997; Nikolic et al., 2020; Zhang et al., 2016).

Resistive gas sensors are promising for wide practical application due to their simple design and low cost, high sensitivity, fast response and the possibility of integration into electronic devices (Nikolic et al., 2020; Neri, 2015; Chai et al., 2022; Korotcenkov et al., 2013). Wide gap semiconductor metal oxides (SMOs) are most often used as the sensitive materials. The analytical signal is formed during the interaction of gas with the surface layer of the semiconductor that leads to a change in resistance (Nikolic et al., 2020; Wang et al., 2010). Significant disadvantages of such sensors include insufficient selectivity and stability, as well as relatively high power consumption caused by high operating temperatures (250–500°C). The development of new strategies for the effective gas detection at close to room temperature (RT) has great potential (Joshi et al., 2018; Majhi et al., 2021a; Srinivasan et al., 2019). Light activation (Espid and Taghipour, 2017; Lee and Yoo, 2022; Wang et al., 2021; Chizhov et al., 2021; Xu and Ho, 2017; Ma Z. et al., 2021; Kumar et al., 2021; Majhi et al., 2021b) allows increasing the concentration of free charge carriers, activating and controlling the kinetics of ongoing chemical processes on the surface of semiconductor oxides, and accelerating the process of desorption of reaction products (Anothainart et al., 2003; Boukhvalov et al., 2021; Liu et al., 2014). This article provides an overview on semiconductor gas sensors operating under illumination and discusses the importance of photoactivation for optimizing gas sensor technology.

## 2 Photoactivated semiconductor metal oxide gas sensor systems

**FIGURE 1:**
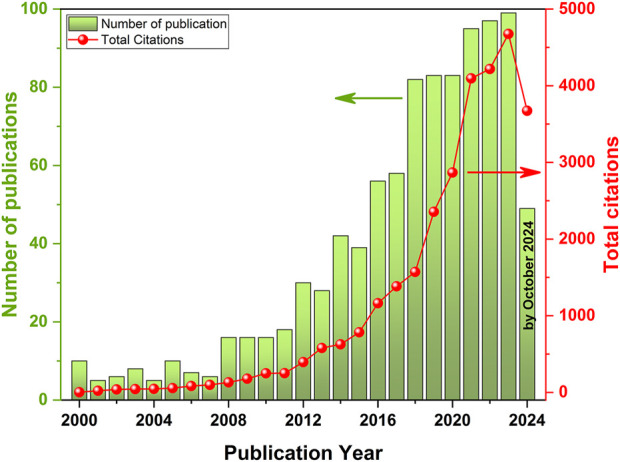
Literature survey of publications for gas sensors operating at RT under photoactivation from 2000 to October 2024, according to Web of Science. Core collection search keywords: TS = gas sensor AND (TS = room temperature OR TS = low temperature) AND (TI = UV OR TI = ultraviolet OR TI = light OR TI = illumination OR TI = LED OR TI = photo OR TI = irradiation OR TI = activation OR TI = photosensitive OR TI = light-activated OR TI = photo-activated).

Over the past decades, the number of scientific publications devoted to the study of photoactivated gas sensors has increased significantly (Figure 1) that demonstrates the effectiveness of this approach in gas detection (Espid and Taghipour, 2017; Wang et al., 2021; Chizhov et al., 2021; Xu and Ho, 2017; Ma Z. et al., 2021; Kumar et al., 2020; Šetka et al., 2021).

The effect of ultraviolet (UV) and visible light irradiation with the energy exceeding band gap of semiconductor/photosensitizer, on the receptor function of wide-gap SMOs is determined by the following factors (Majhi et al., 2021b; Scandola et al., 1994).

• Increasing carrier concentration in a semiconductor matrix. When a semiconductor oxide is illuminated with radiation of appropriate energy for the band gap transition, an electron-hole pair is generated and the electron is excited to the conduction band (CB). The photogenerated hole recombines with an electron localized by oxidizing gas molecules chemisorbed on the surface. Photogenerated electrons increase the concentration of charge carriers in the CB, which provides an increase in the conductivity of the n-type semiconductor. The value of conductivity is determined by the dynamic equilibrium of the processes of adsorption and desorption of an oxidizing gas.
• Changing the type and concentration of surface adsorption sites. Photodesorption leads to a decrease in the concentration of chemisorbed oxygen, whose positions can be occupied by water molecules and hydroxyl groups according to the molecular and dissociative adsorption mechanisms, respectively. This leads to a significant increase in the hydrophilicity of the surface of SMOs under UV radiation.
• Formation of highly active radical particles. Upon detection of volatile organic compounds photolysis of analyte molecules can occur on the semiconductor’s surface that facilitates their subsequent oxidation with chemisorbed oxygen, leading to a change in the conductivity of the semiconductor.
• Decreasing the intergrain energy barrier height by changing the intergrain charge states and increasing the probability of tunneling through the intergrain barriers by decreasing the depletion layer widths in the adjacent grains for polycrystalline materials.

The mechanism of sensor signal formation on the surface of the sensitive layer of resistive gas sensors during photoactivation (UV irradiation in the case of pristine SMOs) can be explained on the basis of ionosortpion concept (Das et al., 2022; Ji et al., 2019; Staerz et al., 2022; Mishra et al., 2004; Mall et al., 2018). In air, oxygen molecules can be adsorbed on the surface of a semiconductor oxide in the form of $O_{2}^{-}$ , $O^{-}$ and $O^{2-}$ ions by capturing electrons from the conduction band (Barsan and Weimar, 2001; Barsan et al., 1999; Barsan et al., 2007). As a result, an electron depletion layer comparing to the bulk is formed. The difference between work functions leads to the formation of a Schottky potential barrier at the boundaries of the semiconductor crystalline grains resulting in the resistance increase. These chemisorbed oxygen species play a key role in interactions with reducing gases. At RT photogenerated holes can recombine with the electrons localized by chemisorbed oxygen ions, facilitating oxygen desorption (Equation 1) while photogenerated electrons can interact with an oxygen molecule to form photosorbed oxygen species (Equation 2) (Chizhov et al., 2023).

$$O_{2\left(ads\right)}^{-}+h^{+}\left(h\nu \right)↔\;O_{2\left(gas\right)}$$ (1)

$$O_{2\left(gas\right)}+e^{-}\left(h\nu \right)↔\;O_{2\left(ads\right)}^{-}$$ (2)

**FIGURE 2:**
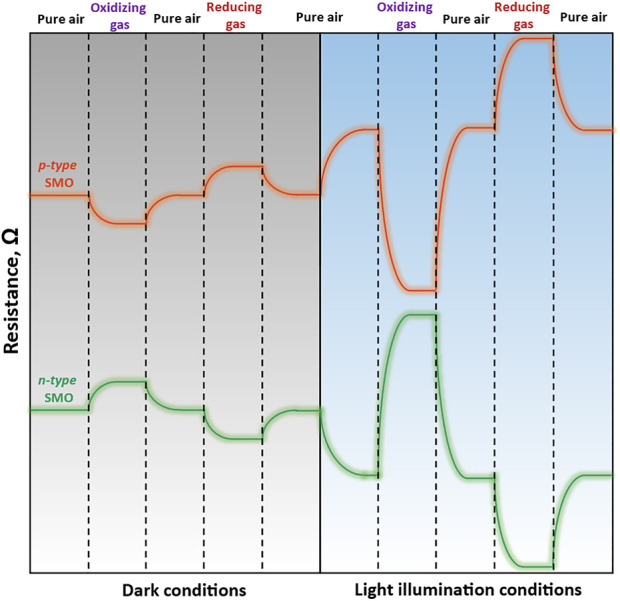
Schematic illustration of SMOs gas sensing response in the presence of oxidizing and reducing gases in dark conditions and under illumination.

The polarity of the sensor response depends on the type of gas-analyte and the main charge carriers. Figure 2 shows a schematic representation of n-type (SnO_2_, In_2_O_3_, ZnO, WO_3_) and p-type (Co_3_O_4_, NiO, CuO) SMOs resistance behavior in the presence of oxidizing (NO_2_, O_3_, Cl_2_) and reducing (NH_3_, CO, H_2_S) gases in dark conditions and after under light irradiation. When n-type SMO interacts with oxidizing gases with higher electron affinity (E_ea_ (NO_2_) = 2.27 eV, E_ea_ (O_3_) = 2.10 eV) then that for O_2_ molecules (E_ea_ (O_2_) = 0.44 eV) (Zhou et al., 2001; Zemke et al., 1972), the resistance increases due to electron depletion layer extension. The adsorption of reducing gases, on the contrary, leads to a decrease in resistance due to their reaction with chemisorbed oxygen and releasing electrons into CB. Under illumination, electron-hole pairs are generated that leads to an increase in the concentration of electrons and photodesorption of oxygen from the surface by photogenerated holes (Equation 1). The consequence of these processes is a decrease in the resistance below the baseline value in a pure air atmosphere. Oxidizing gases will be adsorbed at a higher rate under such conditions, increasing the resistance of the sensor. The amplitude of the decreasing resistance when interacting with reducing gases is smaller, since the reaction requires chemisorbed oxygen, which undergoes photodesorption.

SMOs with p-type conductivity react in a conversely manner. These materials have higher baseline resistance comparing to n-type SMOs due to the less-conducting core and semiconducting hole-accumulation shell on the particles’ surface. Their resistance decreases in the atmosphere of oxidizing gases since gas molecules capture the electrons resulting in the increase in holes concentration. When reducing gas interacts with chemisorbed oxygen species the electrons release into the CB and the hole concentration decreases leading to the increase in resistance. Light illumination can further enhance sensing effect due to availability of more charge carrier species.

### 2.1 Light activation of pristine SMOs

Saura (1994) found that UV illumination could improve the sensitivity of SnO_2_-based gas sensor towards acetone and trichloroethylene by increasing the concentration of photogenerated charge carriers and decreasing the intergranular barriers height. Later E. Comini et al. achieved an increase in response of SnO_2_ thin film gas sensors toward NO_2_ under UV light by concentrating the light flux onto the sensor surface using optical fibers (Comini et al., 2001). These results were supported by Mishra et al. (2004) who proposed a theoretical model of SMOs sensor signal under UV irradiation. They concluded that the sensitivity of sensors based on polycrystalline materials should increase with increasing light fiux density and should decrease with an increase in grain size of the sensor material. A comparative study of single crystal SnO_2_ nanowires when detecting NO_2_ under UV photoactivation and in dark conditions demonstrated high sensor response in the first case. The results showed that UV irradiation is a good alternative to thermal heating not only for activation of surface chemical reactions and products desorption, but also for increase in the sensor response towards oxidizing gases (Prades et al., 2009).

A comparative assessment of the efficiency of porous TiO_2_ and ZnO gas sensors when detecting formaldehyde and acetone at RT under UV light demonstrated that the responses of ZnO were 1,000 times less than the responses of TiO_2_. Such a huge difference between TiO_2_ and ZnO was explained by the quantity of chemisorbed oxygen species on the sensors surfaces under UV light confirmed by the ratio of light and dark currents for these materials (Chen et al., 2012). Additionally, mass-spectrometry analysis confirmed the possibility of oxygen photoadsorption on SMOs under UV illumination (Chizhov et al., 2022). As radiation sources, low-power miniature LEDs have certain advantages (Nam et al., 2024). Replacing UV radiation with a visible light can further reduce energy consumption. Moreover, the visible range of the spectrum accounts for the maximum intensity of solar radiation, which can be additionally used as a light source. It is worth noting that wide gap SMOs are optically transparent in the visible spectral range (Soler-Fernández et al., 2024).

Various approaches can be used to improve the selectivity and reduce the power consumption of SMOs based resistive gas sensors by controlling SMOs morphology, microstructure, type and concentration of bulk defects and surface active sites (Nikolic et al., 2020; Cho and Park, 2016). A promising direction is the creation of new optimized gas sensitive materials that absorb visible light. This approach opens up new possibilities and advantages for practical application. Firstly, visible range light sources (mainly low-power miniature LEDs) are currently widely available and inexpensive, unlike sources with high photon energy (UV range), which are still technologically and materially more expensive. Moreover, such sources consume less energy than UV sources. Secondly, as an alternative, natural sunlight can be used, a significant part of which falls in the visible range (∼5% ultraviolet, ∼43% visible and ∼52% IR range). Shifting the SMOs optical sensitivity to the long-wavelength region is possible by creating defects in their crystal structure (Zhang et al., 2017) or modifying their surface with photosensitizers (Bignozzi et al., 1995).

Commercial gas sensors used until the early 2000’s had an energy consumption of about 400 mW–1 W. They were replaced by smaller sensors with a thin film structure that consume 120–280 mW. The next step in the evolution are the sensors based on MEMS (Micro Electro Mechanical Systems) technology, which are currently considered the most energy efficient with a power consumption of 15–60 mW (Burgués and Marco, 2018). Recent studies have shown ultra-low power consumption for sensors integrated with μLEDs (micro light-emitting diodes) operating under illumination. This method can reduce energy consumption up to several hundreds of microwatts (Cho et al., 2020; Lee K. et al., 2023).

### 2.2 Photosensitization of SMOs

An increase in the SMOs sensitivity and selectivity when detecting various gases under visible light can be achieved using photosensitizers – metal nanoparticles with the plasmon resonance effect (Rossi et al., 2019; Zhang Q. et al., 2018; Gogurla et al., 2014), organic dye molecules (Paolesse et al., 2017; Zhang et al., 2015; Peng et al., 2011) or narrow gap semiconductors of the A^II^B^VI^ (CdS with E_g_ = 2.4 eV; CdSe with E_g_ = 1.7 eV) and A^III^B^V^ groups (InP with E_g_ = 1.35 eV) (Chizhov et al., 2016; Geng et al., 2016; Vasiliev et al., 2013; Yang et al., 2014).

Plasmon resonance (PR) is known as the phenomenon of collective oscillation of valence electrons relative to the atomic core level, coinciding in frequency with external light radiation (Rossi et al., 2019; Khurgin, 2019; Basova et al., 2016). The most mentioned type of sensitizers exhibiting the PR effect under visible light are gold and silver nanoparticles possessing high extinction coefficient and stability (Suh et al., 2021; Panayotov and Morris, 2016). By varying the size and shape of nanoparticles, their extinction coefficient can be controlled over a fairly wide spectral range (Lu et al., 2009; Linic et al., 2011). The photosensitization by plasmonic nanoparticles may be associated with various effects (Chizhov et al., 2021; Rossi et al., 2019; Manjavacas et al., 2014; Kim et al., 2018; Zhang Y. et al., 2018; Ma Q. et al., 2021; Watanabe et al., 2006).

• The PR effect causes the formation of inhomogeneous electric field near metal nanoparticles, which can sufficiently influence on the formation of electron-hole pairs in a semiconductor.
• The multiple scattering of photons by plasmonic nanoparticles can increase the optical path of light.
• Plasmonic nanoparticles can be a source of local heat generation.

In work Zhang Q. et al. (2018) the authors obtained heterostructures based on polycrystalline ZnO and Ag nanoparticles. Compared with pure ZnO, the ZnO/Ag heterostructures show an increase in the sensor response towards NO_2_ (0.5–5 ppm) at RT under photoactivation in 365–520 nm spectral range. The maximum signal and the greatest selectivity were achieved at λ = 470 nm corresponding to the plasmon resonance of Ag nanoparticles. Encapsulation of Ag nanoparticles in zeolitic imidazolate frameworks-8 (ZIF-8) is another strategy for increasing sensitivity by combining high porous material with photoactive particles. The crucial role of the Ag nanoparticles size effect on triethylamine sensing was shown: the less the particles’ size the better effect of plasmon resonance realized, resulting in higher sensor signal (Yao et al., 2024).

Decoration of ZnO with Au nanoparticles can also lead to significant enhancement of gas sensing parameters. Thus, combination of several effects, like localized surface plasmon in Au nanoparticles, spillover effect and the enhancement of chemisorption and dissociation of gas results in the enhanced sensor response of the Au-modified ZnO nanosheet sensor to NO_2_ at RT (Mun et al., 2013). Sensitivity of Au-ZnO nanocomposite can also be enhanced both in UV and visible region, leading to detection of NO (Gogurla et al., 2014). Composite systems modified with bimetallic clusters, like AuAg/ZnO (Sun et al., 2024a) and Pd@Ni/ZnO (Sun et al., 2024b), showed superior CH_4_ sensing performance at RT under UV illumination.

Organic photosensitizers – organometallic complexes, are of particular interest because the central cation can be an active adsorption or catalytic center in solid-gas interaction, providing an increase in selectivity. Under suitable radiation, the organometallic complex goes into an excited state, the electrons first move from the highest occupied molecular orbital (HOMO) to the lowest unoccupied molecular orbital (LUMO), from where they can be transferred to the semiconductor’s CB. Photogenerated holes remaining at the HOMO level can drift to the crystal under electric field gradient and subsequently recombine with electrons localized on chemisorbed molecules of oxidizing gases. Thus, visible light irradiation of hybrid materials leads to the photodesorption of oxidizing gases (Anothainart et al., 2003).

To increase the efficiency of detecting gases under visible light using hybrid materials, a number of requirements are imposed on an organic photosensitizer (Kaushik et al., 2015).

• high molar extinction coefficient in the spectral region coinciding with the LED radiation energy;
• chemical stability, thermal stability and photostability towards probe analytes and external conditions;
• optimal alignment of energy levels to ensure energetically favorable charge transfer: the LUMO of the photosensitizer should be higher in energy than semiconductor’s CB, and the HOMO should be between the semiconductor’s CB and the oxidation reaction potential;
• optimal arrangement of the photosensitizer molecular parts in space: HOMO should be far away from the semiconductor surface, LUMO should be on the ligand or fragment of the molecule associated with the semiconductor surface.

A number of works show effective results on using organic molecules as photosensitizers. A study of the gas sensor properties of hybrid materials with heterocyclic Ru(II) complexes towards NO or NO_2_ in pulsed illumination mode with different wavelengths (470, 525 and 630 nm) showed that these photosensitizers allow shifting spectral sensitivity to the visible region and detecting low concentrations of nitrogen oxides at RT (Rumyantseva et al., 2018; Nasriddinov et al., 2020). Different composites based on porphyrins coated SMOs were developed, presenting promising application in the detection of VOCs (Sivalingam et al., 2013; Ekrami et al., 2018; Magna et al., 2017; Magna et al., 2014).

The authors Chizhov et al. (2016) studied the effect of the surface sensibilization of nanocrystalline ZnO, SnO_2_ and In_2_O_3_ with CdSe colloidal quantum dots (QD) on the interaction with NO_2_ at RT under green light (λ = 525 nm) corresponding to the CdSe QD absorption band. Photogenerated electrons are injected from the QD into the SMOs CB, while photoexcited holes can go into recombination with electrons localized in chemisorbed O_2_ and NO_2_ species. Besides, the photogenerated electrons enhances the interaction with the oxidizing gas NO_2_. The most effective photosensitization was demonstrated for the In_2_O_3_-based nanocomposites due to the maximum difference in the position of the energy level of the photoexcited electron at the CdSe QD and the bottom of the In_2_O_3_ conduction band.

In addition to all of the above materials, in work Wang et al. (2024) Bi_2_WO_6_ nanosheets supported on CuBi_2_O4 nanorods were used as gas sensors working at 110°C under blue light. Formation of heterojunction allowed increasing sensitivity and selectivity for n-butyl alcohol among 25 kinds of common organic gases. The authors Liu et al. (2024) obtained heterostructures based on mesoporous Cu-BTC MOF (tricarboxylic acid metal organic framework) and Bi_2_MoO_6_ nanocrystals. Such materials showed high selectivity when detecting acetone under UV light. Some other materials, like AgMoS_2_ and PdMoS_2_ (Rawat et al., 2024), Co_y_Zn_1-y_Fe_2_O_4_ (Ravikumar and Sivaperuman, 2024) showed enhanced light-assisted sensing response towards NH_3_ at RT.

Van der Waals two-dimensional (2D) materials and heterostructures, such as graphene, transition metal dichalcogenides, MXenes also have demonstrated considerable results for gas detection under photoactivation (Joshi et al., 2018; Kumar et al., 2020; Deb et al., 2024). Herein, several examples can confirm their feasible practical application for gas sensing: g-C_3_N_4_ modified ZnO composite for CH_4_ detection (Zhang et al., 2023), Ti_3_C_2_T_x_/TiO_2_/graphene composite with sandwich structure for NH_3_ sensing (Lin et al., 2024), In_2_O_3_/rGO composites for NH_3_ detection in humid conditions (Nasriddinov et al., 2023), Bi_2_S_3_/Sb_2_S_3_ heterostructure for trace H_2_S detection (Yu et al., 2024), graphene-wrapped ZnO nanocomposite with enhanced toluene sensing (Huang et al., 2024). Theoretical analysis of the HCHO adsorption behavior on graphenylene-like ZnMgX_2_ (X = O, S) monolayers showed that this type of interaction results in a strong chemisorption and does not require the presence of dopant (Chang et al., 2024). Significant improvements in gas sensor characteristics by 2D materials may be associated with tunable band gaps, unusual electronic and optical characteristics, formation of p–n heterojunctions, which lead to effective charge separation.

## 3 Further development prospects

The creation of gas sensors working under photoactivation is a new, actively developing direction, which has a huge potential for the design of breakthrough sensor technologies. The use of photoactivated gas sensors with low power consumption and compact LEDs allows one to create miniature monitoring systems and portable gas analyzers (electronic noses) not only for indoor and outdoor air monitoring, but also in other areas including breath analysis for non-invasive medical diagnostics (Zheng and Cheng, 2019; Righettoni et al., 2015). Such tasks impose strong requirements for sensitivity and stability of gas sensors, since the concentration of gases in the exhaled air lies in the ppb range, and humidity reaches up to 100% (Güntner et al., 2019). Compared with chromatographic and spectroscopic methods, the electronic nose systems are cost-effective and allow for large-scale and rapid research (Park et al., 2019; Hu et al., 2019; Lee SW. et al., 2023; Eranna et al., 2004). Another promising application of the sensors is robotics, which will be controlled by artificial intelligence system (Chen et al., 2019). Robots similar to humans are being created now, so they need to use some kind of sensor organs close to human ones (including an artificial nose), and automatic centralized control of Smart Home systems is no longer possible without such sensors (Park et al., 2019; Kim et al., 2021; Jeong et al., 2020). An energy-efficient gas sensor integrated into an unmanned aerial vehicle (drone) will increase its battery life for monitoring the air condition in industrial cities and hard-to-reach areas with a high level of gas pollution (Rohi et al., 2020). The further works have to be done to integrate the electronic nose and electronic tongue systems into smartphones (Oletic and Bilas, 2013; Hasenfratz et al., 2012; Aydogmus et al., 2023). The general trend in the development of new sensors and organ-on-a-chip technology is to make them more and more miniature and energy-efficient (Majhi et al., 2021a; Suh et al., 2021; Burgués and Marco, 2018; Leung et al., 2022; Low et al., 2021; Ingber, 2022).

However, an understanding of the effect of light irradiation on the SMOs sensor signal has been attained only in the case of oxidizing gases (NO_2_, O_3_) detection, while the vast majority of target substances belong to reducing gases of various chemical nature. To achieve the necessary characteristics of photoactivated sensors, fundamental research is needed: development of new materials with high gas sensitivity at close to room temperature under UV or visible light; investigation of the reactivity of photo- and gas-sensitive materials in interaction with reducing gases; investigation of the sensor properties under conditions that meet the real practical problems of air monitoring.

## 4 Concluding remarks

In summary, this review discussed recent progress in light-activated gas sensors and illustrated that significant tasks still need to be done to address the shortcomings in the future. Wide gap semiconductor oxides can be used as a functional structure, which provides economic advantages, mass production and high stability. On the other hand, the strategy of replacing thermal activation with light irradiation realizes not only high-performance gas detection at close to room temperature, but also facilitates the development and production of portable, integrated, flexible and multifunctional sensing devices and IoT applications (Nikolic et al., 2020; Soler-Fernández et al., 2024; Cho et al., 2018). However, there are still some limitations and disadvantages in design and manufacture of light-activated composites for gas sensor systems. These pitfalls must be taken into account before a full-scale transition to a new technological production: the lifetime of the sensitive layer, especially organic dyes that tend to fade under the influence of light over the time; selectivity towards certain gases; stability in a humid atmosphere; the possibility of regenerating the sensitive layer without heating.

The negative effects of humidity can be eliminated by using porous membrane materials, zeolites, metal-organic frameworks. Such passive filters operate on a dimensional effect; pores of a certain size can only allow certain gas molecules to pass through or interact with analytical gases using the “guest-host” mechanism. Modification of organic dye molecules to obtain more stable and effective structures or its encapsulation can enhance their persistence. The use of catalytic overlayers, different additives and modifiers can further increase selectivity for detection of certain group of gases due to electronic and chemical sensitization effects. These approaches are expected to improve sensor characteristics for gas detection at room temperature and expand the scope of practical application.
